# Expression and Function of Hypoxia Inducible Factor-1*α* and Vascular Endothelial Growth Factor in Pulp Tissue of Teeth under Orthodontic Movement

**DOI:** 10.1155/2015/215761

**Published:** 2015-09-09

**Authors:** Fulan Wei, Shuangyan Yang, Hui Xu, Qingyuan Guo, Qi Li, Lihua Hu, Dongxu Liu, Chunling Wang

**Affiliations:** ^1^Department of Orthodontics, School of Stomatology, Shandong University, Jinan, Shandong, China; ^2^Shandong Provincial Key Laboratory of Oral Tissue Regeneration, No. 44, Wen Hua Xi Lu, Shandong, Jinan 250012, China; ^3^Department of Orthodontics, School of Stomatology, Capital Medical University, Beijing 100050, China

## Abstract

Orthodontic force may lead to cell damage, circulatory disturbances, and vascular changes of the dental pulp, which make a hypoxic environment in pulp. In order to maintain the homeostasis of dental pulp, hypoxia will inevitably induce the defensive reaction. However, this is a complex process and is regulated by numerous factors. In this study, we established an experimental animal model of orthodontic tooth movement to investigate the effects of mechanical force on the expression of VEGF and HIF-1*α* in dental pulp. Histological analysis of dental pulp and expressions of HIF-1*α* and VEGF proteins in dental pulp were examined. The results showed that inflammation and vascular changes happened in dental pulp tissue in different periods. Additionally, there were significant changes in the expression of HIF-1*α* and VEGF proteins under orthodontic force. After application of mechanical load, expression of HIF-1*α* and VEGF was markedly positive in 1, 3, 7 d, and 2 w groups, and then it weakened in 4 w group. These findings suggested that the expression of HIF-1*α* and VEGF was enhanced by mechanical force. HIF-1*α* and VEGF may play an important role in retaining the homeostasis of dental pulp during orthodontic tooth movement.

## 1. Introduction

It has been known that because of application of mechanical force to the tooth crown a tooth can be moved to a desirable spot. The transmission of the force to alveolar bone is mediated by the response of periodontal ligament (PDL), which leads to adaptation of the periodontal tissues to mechanical stress [1]. The tissue response caused by orthodontic force does occur not only in periodontal tissue but also in dental pulp tissue. Orthodontic force can cause the dental pulp circulatory disturbances and vascular changes [2, 3], which caused the oxygen levels descending in dental pulp; actually it represented a process of inflammation [4, 5]. As a pathological stimulus, hypoxia will inevitably induce the defensive reaction of dental pulp tissue so as to maintain the stability of internal environment [6, 7]. Previous studies indicated that pulp anaerobic can lead to the formation of new blood vessels in the process of orthodontic tooth movement [1]. Angiogenesis, the formation of new blood vessels, is a complex process including extracellular matrix remodeling, secretion of proteolytic enzymes, endothelial cell migration and proliferation, capillary differentiation, and anastomosis [8]. A number of cytokines and growth factors were implicated in angiogenesis.

Hypoxia inducible factor-1 (HIF-1), a heterodimeric transcription factor, is composed of *α* (inducible) and *β*- (ubiquitous-) subunits [9]. HIF-1*α* is stabilized and translocated to the nucleus under hypoxic conditions. *β*-subunit protein is constitutively present, whereas the stability and transcriptional activity of *α*-subunit are precisely controlled by the intracellular oxygen concentration [10, 11]. Recent studies showed that HIF-1 mediates the angiogenic response to hypoxia through upregulating the expression of multiple angiogenic factors including VEGF and angiopoietins [12]. VEGF is involved in both angiogenesis and osteogenesis which serves various biological functions, such as increasing vascular permeability, promoting monocytic chemotaxis, and regulating endochondral ossification [13, 14]. It was found that when VEGF was inactivated in mice, blood vessel invasion was nearly abolished and trabecular bone formation was impaired [15]. VEGF is regarded as the most essential factor for the differentiation of the vascular system among all the proangiogenic factors [16]. These observations provide direct evidence that VEGF plays an important role in bone formation and orthodontic tooth movement.

According to previous study, we found that HIF-VEGF signaling pathways might be involved in response of dental pulp tissue to mild continuous mechanical force* in vitro* [17]. In order to analyze the expression and function of HIF-1*α* and VEGF in dental pulp* in vivo*, we established an animal model of tooth movement to explore how the expression of HIF-1*α* and VEGF is affected by stimulating pulp hypoxia in pulp tissue after orthodontic force and to probe into the possible mechanism of pulp tissue to maintain their own stability in the process of orthodontic tooth movement.

## 2. Materials and Methods

### 2.1. Animals

The study design was submitted to and approved by the Animal Ethical Committee of Shandong University (number ECAESDUSM2012075).

This study contained forty-five male Wistar rats, eight weeks of age, with an average weight of 250 ± 20 g, obtained from School of Stomatology, Shandong University. Animals were fed with a standard diet (Vital River Laboratory Animal Company, Beijing, China) and mineral water* ad libitum* to avoid any discomfort after the orthodontic appliance inserting. Rats were anesthetized with an intraperitoneal injection of 2.5% tribromoethanol, 0.25 g/kg body weight. The experimental groups received a nickel-titanium closed coil spring (0.008′′  ×  0.032′′), placed from the right maxillary first molars to the incisors of animals. We prepared a cervical groove on the incisors, in which the ligature wire was seated and secured with light-cured resin (Z100, 3M, Sumaré, São Paulo, Brazil). This coil spring was employed for mesial inclination of the first molar. According to previous studies, 40 or 50 g force was applied in their experimental rat model [18, 19]. However, we thought that a force of 40 or 50 g appears too high, so we applied a force of 30 g in our experiment.

The animals were randomly divided into six groups including one control group not submitted to force application and five experimental groups of 1, 3, 7, 14, and 28 d of force application.

### 2.2. Tooth Movement Measurements

Animals were killed at each experimental time point and the maxillae were isolated. The tooth movement was measured between the distal surface of the first molar and the mesial surface of the second molar using a vernier caliper with a minimum measurable distance of 0.05 mm. All measurements were performed three times by one operator. Take mean value as a tooth movement distance.

### 2.3. HE Staining

After the experimental period, the animals were sacrificed by a transcardial perfusion of 4% paraformaldehyde fixing solution. The maxilla was sectioned in the area of the maxillary first molars and placed in the same fixative for 48 hours. Subsequently, the fixed tissues were decalcified in 10% EDTA solution for 1 month. After completion of decalcification, the blocks of tissue which contain first molars and surrounding alveolar bone were dehydrated in graded alcohol. Then the specimens were embedded in paraffin and serial longitudinal 5 *μ*m thick sections were cut in a mesiodistal direction, parallel to the long axis. The sections were stained with HE and examined under light microscopy for analyzing the pulp structures and vascular changes. Photomicrographs were obtained.

### 2.4. Immunohistochemistry

To investigate the protein level of HIF-1*α* and VEGF, immunohistochemistry analysis was used. Immunostaining was performed according to the Abcam System. Sections were deparaffinized in xylene and hydrated in graded alcohol. To block endogenous peroxidase activity, the sections were incubated in 3% H_2_O_2_ in absolute methanol for 20 min. Then the sections were permeabilized in PBS, using normal goat serum for 20 min to block nonspecific binding. After rinsing, sections were incubated with monoclonal mouse anti-HIF-1*α* (1 : 200, Abcam Inc., Cambridge, MA), monoclonal mouse anti-VEGF (1 : 200, Abcam Inc., Cambridge, MA). Incubation was in a humid chamber at 37°C overnight. Next, sections were incubated with biotinylated goat anti-mouse IgG (1 : 200, Zhongshan Golden Bridge Biological Technology Co. Ltd., Beijing, China) for 2 h at room temperature. The signals were visualized using 3-3′ diaminobenzidine (DAB, Wako, Osaka, Japan) and lightly counterstained with hematoxylin. For negative controls, specimens were stained without primary antibodies.

### 2.5. Image Acquisition

All specimens were analyzed under bright-field microscopy (BX51, Olympus, Tokyo, Japan), and images were captured with a CCD camera (CS600, Olympus, Tokyo, Japan).

### 2.6. Statistical Analysis

Data are presented as mean and standard deviation (mean ± SD). Both descriptive and inferential statistical analyses were used to analyze the protein level of HIF-1*α* and VEGF.

## 3. Results 

### 3.1. Tooth Movement

As shown in Table 1, the distance of tooth movement increased with the extension of experimental period. The highest mean value of tooth movement was found in the 4 w group.

### 3.2. Histology

Histological examination showed that tooth movement for different periods presented different changes in pulp tissue structure, as demonstrated by cellular and structural events coupled with inflammatory process. Our study revealed the presence of inflammatory cells throughout the experimental period, indicating an inflammatory process as a response to orthodontic force. We could observe the inflammatory cells inside the blood vessels and in the pulp connective tissue, such as neutrophils and monocytes, which act as phagocytes and restrict the inflammatory process. Also the edema at pulp tissue was observed in the experimental groups.

**Figure 1:**
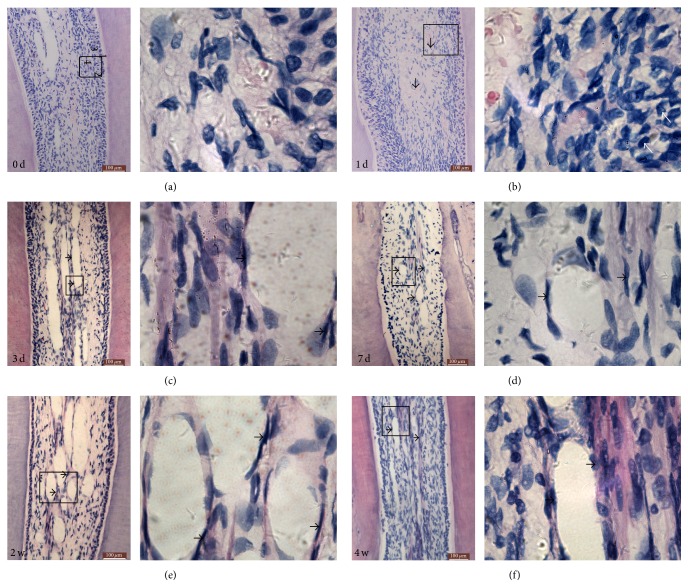
Photomicrograph of histological section of the dental pulp for different time periods. (a) Showing the control group: 1 = odontoblastic layer; 2 = Weil zone (cell-free zone); 3 = cell-rich zone; 4 = central pulp area. (b) Photomicrograph of a histological section showing in detail the odontoblastic layer of the dental pulp of the 1 d group. White arrows indicate the inflammatory cells; black arrows indicate the areas of congested vessels. (c) Photomicrograph of a histological section showing areas of congested vessels and edema in the pulp center of the 3 d group. Arrows indicate the areas of congested vessels. (d) Photomicrograph of a histological section showing areas of congested vessels and edema in the pulp center of the 7 d group. Arrows indicate the congested vessels. (e) Photomicrograph showing the vascularization of the central layer of the pulp of the 2 w group. Arrows indicate the vessels. (f) Photomicrograph showing blood cells inside the vessels of the pulp of the 4 w group (images on the left: magnification ×200, bar = 100 *μ*m; images on the right: magnification ×1000).

In the control group, the odontoblasts were adjacent to the predentin layer, with the cell bodies around the pulp and the cytoplasm crossing the predentin layer and reaching the dentinal tubules (Figure 1(a)). Compared to control group, the odontoblasts maintained their characteristic organization in all experimental groups, but the nuclei had expanded and diffused chromatin and the cytoplasm was more basophilic, with sparser cells (Figures 1(b)–1(f)). This alteration occurred mainly in the mesial surface of the pulp of the teeth. At the same time, in the control group, we can observe a cell-rich zone interiorly to this cell-free zone which was very clear on the coronal pulp (Figure 1(a)).

In the 3 d group, the pulp showed normal characteristics, but with changes in the odontoblastic layer in the mesial surface of the coronal pulp (Figure 1(c)). The central area of the coronal pulp displayed the Weil zone in the control group, which is the cell-free zone at the periphery of the dental pulp, while in the experimental groups the Weil zone disappeared at the areas in which the odontoblastic layer was more altered obviously and with larger cells. The pulp tissue swelled and appeared more loose as if some serous fluid accumulated excessively between connective-tissue cells. At 1 d and 3 d of tooth movement, the odontoblasts were exhibited normally and the Weil zone was once again normally observed on the coronal pulp (Figures 1(b) and 1(c)). In experimental groups, the cell-rich layer was larger than the control group, with characteristic cells such as fibroblasts, undifferentiated mesenchymal cells, and defense cells. These cells were distributed homogeneously, with a slight increase around blood vessels.

Vascularization in the control group was concentrated in the central zone of the coronal and root pulp, characterized by the presence of wider vessels (Figure 1(a)). In the experimental groups, vascularization was also concentrated in the central region of the dental pulp and characterized by the presence of wider blood vessels, and it was more closely to the odontoblastic layer. The 1 d and 3 d groups exhibited areas of congested vessels in the central region of the pulp especially at the root, characterized by the presence of erythrocytes (Figures 1(b) and 1(c)). The vessels were still congested at 2 w and often displayed a substance with light purple staining, suggesting the presence of plasma proteins. In the control group and 3 d experimental group we also observed accessory root canals especially at the furcation region of the molars (Figures 1(a) and 1(c)). These root canals contained blood vessels, nerves, and collagen fibers and allowed contact between the pulp tissue and the connective tissue in the periodontal ligament. The regions of accessory root canals presented a sign of inflammatory reaction at the initial periods of tooth movement.

### 3.3. Immunohistochemistry Examination

Immunohistochemical analysis showed that the expression of HIF-1*α* and VEGF proteins changed obviously under orthodontic force.

**Figure 2:**
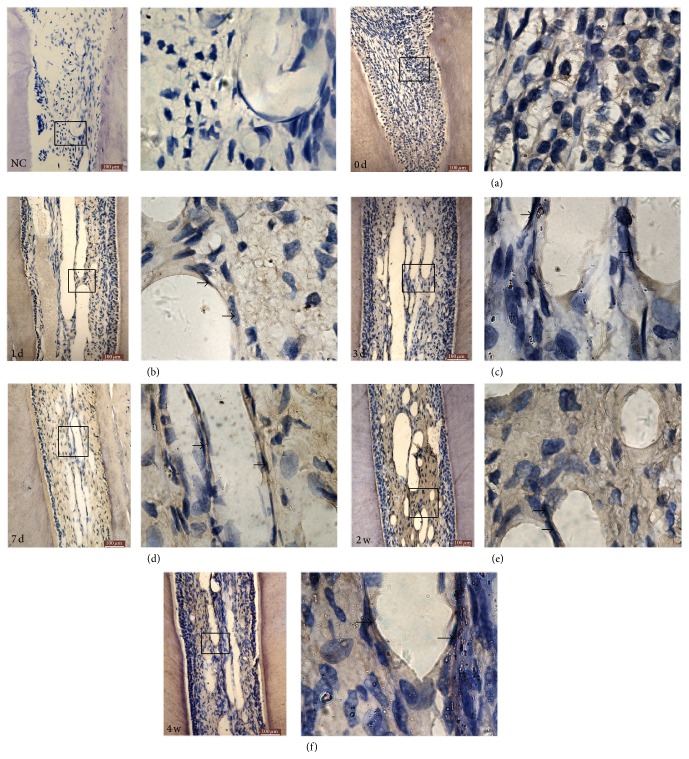
Immunohistochemistry analysis of HIF-1*α* protein expression in rat dental pulp tissue after orthodontic force. HIF-1*α* protein was weakly stained in control group (a). (b–e) HIF-1*α* protein expression became strongly stained after 1, 3, and 7 d of orthodontic force and reached the peak level at 2 w group. Then HIF-1*α* protein expression weakened in the 4 w group but was still higher than the control group (f). Arrows indicate the vessel wall (images on the left: magnification ×200, bar = 100 *μ*m; images on the right: magnification ×1000).

HIF-1*α* protein was poorly stained in dental pulp of control group (Figure 2(a)). Yet, staining of HIF-1*α* was markedly positive in the 1, 3, 7 d, and 2 w group; then it weakened in 4 w group (Figures 2(b)–2(f)). The expression of HIF-1*α* was mainly detected in the nuclei of odontoblasts, pulpal fibroblast, vascular endothelial cells, and mononuclear macrophage under mechanical force. HIF-1*α* was also observed in the cytoplasm of these cells. In the 1 d and 3 d groups, HIF-1*α* expression was slightly stronger compared to the control group. In the 7 d group, expression of HIF-1*α* was stronger than the 3 d group. In the 2 w group, HIF-1*α* was strongly expressed in the nuclei and cytoplasm of odontoblasts, pulpal fibroblast, and vascular endothelial cells. The strongest expression of HIF-1*α* in the nuclei during the whole experiment was detected in this group. In the 4 w group, HIF-1*α* was expressed slightly compared to the 2 w group but was stronger compared to the control group.

**Figure 3:**
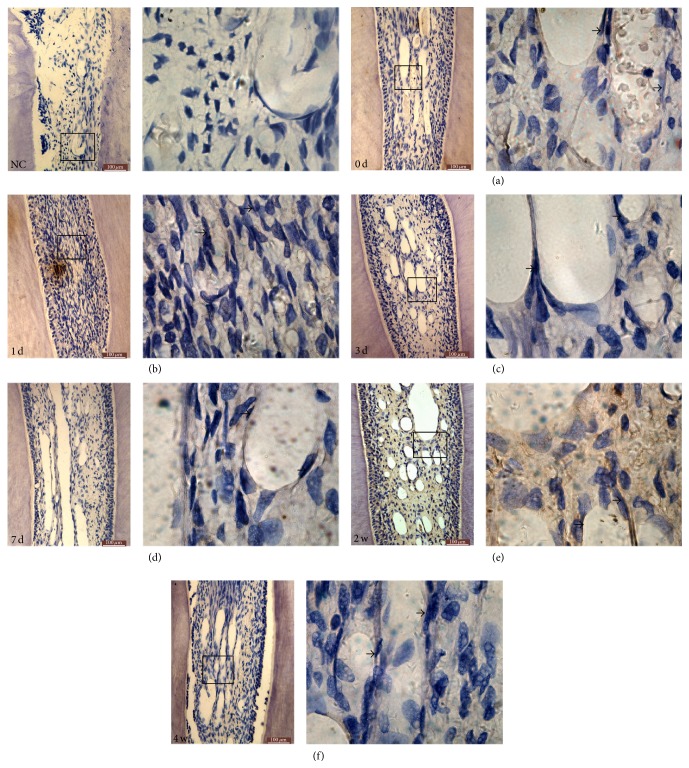
Immunohistochemistry analysis of VEGF protein expression in rat dental pulp tissue after orthodontic force. VEGF protein was weakly stained in control group (a). VEGF protein became slightly stained after 1 d of orthodontic force (b). (c–e) VEGF protein expression became positively stained after 3 d, 7 d, and 2 w after orthodontic force and also reached the peak level at 2 w group. VEGF protein expression then declined in the 4 w group (f). Arrows indicate the vessel wall (images on the left: magnification ×200, bar = 100 *μ*m; images on the right: magnification ×1000).

On the other hand, VEGF immunostaining was observed in newborn vascular endothelial cells in the control group (Figure 3(a)). Then VEGF staining was lightly colored in 1 d group (Figure 3(b)). Subsequently, VEGF staining became stronger in the 3, 7 d, and 2 w groups and it weakened in the 4 w group (Figures 3(c)–3(f)). The expression of VEGF was mainly observed in vascular endothelial cells, fibroblasts, and macrophages. In the 3 d group, VEGF expression was slightly stronger but there was no change when compared to the control group. Following the same kinetics, VEGF protein expression increased further up to 2 weeks and its levels reached the peak. Finally, the expression of VEGF became weak in 4 w group compared to the 2 w group. In 4 w group, cytoplasmic expression of VEGF was slightly expressed but still stronger than the control group.

## 4. Discussion

Our present study showed significant vascular changes early in the initial time of tooth movement and an inflammatory process in the dental pulp as a response to orthodontic force. We observed the initial inflammatory cells inside the blood vessel in the dental pulp tissue. Under orthodontic force application, the expression of HIF-1*α* and VEGF changed differently to regulate the hypoxic environment in dental pulp.

As we know, the dental pulp is surrounded by a rigid, noncompliant shell and its survival depends on the blood vessels. As a consequence of these unusual environmental constraints, changes in pulpal blood flow or vascular tissue pressure might bring in serious implications for the health of the dental pulp [20, 21]. During the process of tooth movement, periodontal ligament is compressed in the direction of orthodontic force and the dental pulp was also in a state of stress. Orthodontic forces are known to produce inflammatory reactions as well as cell damage and circulatory disturbances in dental pulp tissue [22, 23]; in fact these are a presentation of local inflammation. The circulatory disturbances will lead to the oxygen level descending; thus it creates a hypoxic environment in dental pulp. Then the defensive reaction in dental pulp will happen through stimulating a series of cytokines and signal pathways to regulate the inflammatory reaction and cell damage so that it can maintain the stability of internal environment [6, 7]. In addition, hypoxia is able to induce the inflammation reaction in dental pulp by induction of gene expression in inflammatory cells. The molecular mechanism of gene expression is related to many mediators and factors; HIF-1*α* and nuclear factor-*κ*B (NF-*κ*B) are considered as the main of correlative transcription factors. NF-*κ*B subunits can activate the HIF-1*α* promoter and modulate HIF-1*α* expression [24]. Furthermore, previous study confirmed that hypoxia increased the expression of the proinflammatory cytokines such as interleukin- (IL-) 1*β*, IL-6, IL-8, VEGF, and tumor necrosis factor-*α* (TNF-*α*). These proinflammatory cytokines play an important role in orthodontic tooth movement through regulation of matrix metalloproteinase expression and osteoclast differentiation [25, 26]. In our experiment, we can observe the initial inflammatory cells inside the blood vessels in the pulp tissue at 1 d and 3 d, such as neutrophils, eosinophils, and monocytes. As we mentioned before, it is a hypoxic environment in the initial phase of orthodontic tooth movement, and inflammatory reaction occurred in the dental pulp tissue due to short-term hypoxia. Many studies have demonstrated that hypoxia has impact on the activity of NF-*κ*B [27], while hypoxia-induced NF-*κ*B modulated the levels and activity of HIF-1*α* [24]. On the other hand, some proinflammatory cytokines (TNF-*α*, IL-1, IL-6, and IL-8) and proinflammatory enzymes (cyclooxygenase-2 (COX-2), nitric oxide synthase) are controlled by NF-*κ*B [27]. Also* in vitro* experiments have showed that hypoxic conditions increased the expression of IL-6, IL-8, COX-2, and VEGF in dental pulp cells [28]. However, enhanced immune-reactivity of HIF-1*α* is not only caused by hypoxic conditions, but also normoxic conditions, since the promoter of HIF-1*α* is responsive to NF-*κ*B and the stabilization of HIF-1*α* is also dependent on NF-*κ*B in normoxia [25, 29, 30]. Based on the present results in this field, the function and mechanism of hypoxia in dental pulp tissue during the process of orthodontic tooth movement require further investigation.

Although inflammation is induced by hypoxia, we think that the inflammation may act as a physiological signal for angiogenesis. VEGF, as a proinflammatory cytokine, also has angiogenic activity [31, 32]. Angiogenesis or the so-called vascularization is a process of neovascularization, which is a complex process including extracellular matrix remodeling, endothelial cell migration and proliferation, secretion of proteolytic enzymes, capillary differentiation, and anastomosis [8]. Numerous cytokines and growth factors regulated angiogenesis. VEGF is considered as the most essential factor for the differentiation of the vascular system among all the proangiogenic factors [16]. As previously stated, hypoxia is a strong inducer of VEGF expression [33, 34] and VEGF is regulated by both osterix and HIF-1*α* [35]. It is demonstrated that HIF-1*α* is a major regulator of cellular response to hypoxia [36, 37]. Under hypoxic conditions, HIF-1*α* is in a stable state and is translocated to the nucleus where HIF-1*α* binds to its dimerization partner, HIF-1*β*; then it stimulated the expression of its target genes, such as VEGF [38, 39]. Additionally, our early study indicated that the expression of VEGF increased in dental pulp after application of mechanical force* in vitro* [17]. In our experiment, we have shown that the expression of HIF-1*α* and VEGF changed when they are subjected to hypoxic stress and reached the peak at 2 w under the orthodontic force. So we speculated that in the early period orthodontic movement induced a hypoxic environment and hypoxia leads to the induction and increasing expression of HIF-1*α* while VEGF decreases instead because the blood vessels were closured temporarily. With time increasing, VEGF expression increased which is followed by upregulation of HIF-1*α* because the oxygen level continues descending. In turn, VEGF promotes new blood vessels forming and hence brings various necessary nutrients and enhances the level of oxygen to adapt to the low oxygen environment [40, 41]. Thus the hypoxic situation was relieved and it increased the oxygen supply of dental pulp tissue so as to keep the balance of internal and external environment and establish the tissue homeostasis. In accordance with our findings, the study published by Römer et al. [28] also found upregulation of HIF-1*α* expression in the dental pulp tissue of orthodontic moved teeth. They emphasized the transient immune reactivity of HIF-1*α* during the initial phase of orthodontic treatment in dental pulp. Besides, our results are consistent with previous study, which indicated that HIF-1*α* activated VEGF promoter activity [35] and HIF-1*α* upregulates VEGF so as to enhance bone remodeling [42]. On the other hand, in our study, the force magnitude is certain and appropriate and HIF-1*α* and VEGF presented such changes. However, if the force is too small, it is not enough to cause tooth movement and the expression of HIF-1*α* and VEGF may be negative. In contrast, if the force is too high, it would induce the degeneration and necrosis of dental pulp; thus the expression of HIF-1*α* and VEGF would be another case. Therefore, whether the expression of HIF-1*α* and VEGF is dependent on force magnitude will need to be further gone into.

In summary, we may conclude that tooth movement caused pulpal tissue alterations coupled with an inflammatory process. The expression of HIF-1*α* and VEGF was enhanced by mechanical force, which indicated that HIF-1*α* and VEGF may play an important role in retaining the homeostasis of dental pulp during orthodontic movement. HIF-1*α* may serve as a global mediator of the angiogenic response to hypoxia by inducing multiple angiogenic factors including VEGF.

## Supplementary Material

Histological examination of periodontal tissues.

## Figures and Tables

**Table 1:** Molar tooth movement over the course of time in different groups (mm, mean ± SD).

	0 d	1 d	3 d	7 d	2 w	4 w
Tooth movement (mm)	0	0.13 ± 0.014	0.21 ± 0.024	0.28 ± 0.022	0.52 ± 0.03	1.08 ± 0.04
