# Supplementary material for: Expression and Function of Hypoxia Inducible Factor-1α and Vascular Endothelial Growth Factor in Pulp Tissue of Teeth under Orthodontic Movement
Source: Mediators Inflamm. 2015 Sep 9;2015:215761. doi: 10.1155/2015/215761 (PMC4579319; doi:10.1155/2015/215761)
Supplement: Supplementary file 1 — Histological examination of periodontal tissues. [file 215761.f1.zip › 215761.f1/description of supplementary materials.docx]

Histological examination of periodontal tissues

Histological analysis revealed that periodontal tissues appeared different changes in different periods after application of orthodontic force.

In the control group, periodontal membrane is arranged in line, and the width of the periodontal membrane is normal. The surface of alveolar bone is smooth, and there are no obvious signs of osteogenesis and bone absorption. In 1 d and 3 d group, periodontal membrane became narrow on the pressure side. The surface of alveolar bone was rough and there were resorption lacunae on the bone surface. While periodontal membrane became broad on the tension side and the collagen fibers were stretched and arranged disorderly, with dilated and congested blood vessels.

In the 7 d group, bone resorption lacunae increased, within a large number of multinucleated osteoclasts on the pessure side. Alveolar bone absorption was more obvious. On the other hand, periodontal membrane fibers were disarranged and thickened on the tension side. Cuboidal osteoblasts appear on the alveolar surface of tension zone. In 2 w group, we observed an increase in the number of fibroblasts on the pressure side. Osteoclasts were still visible on the alveolar bone surface, extensive bone resorption was found. Compared to the pressure side, we could see new bone line and osteoblasts which arranged neatly along the surface of the newly formed bone on the tension side. In 4 w group, periodontal tissue was being repaired, new bone deposition was visible in the bone resorption lacunae on the pressure side. On the tension side, we could observe rows of osteoblasts and new bone or osteoid formation.

Figures

Histology of PL in control group and experimental group (HE, 200x)

(ps: pressure side; ts: tension side )
