# Supplementary material for: Expression and Function of Hypoxia Inducible Factor-1α and Vascular Endothelial Growth Factor in Pulp Tissue of Teeth under Orthodontic Movement
Source: Mediators Inflamm. 2015 Sep 9;2015:215761. doi: 10.1155/2015/215761 (PMC4579319; doi:10.1155/2015/215761)
Supplement: Supplementary file 1 — Histological examination of periodontal tissues. [file 215761.f1.zip › 215761.f1/mat.215761.v2.docx]

**Supplementary materials**

Histological examination of periodontal tissues

**Control group**: The periodontal membrane is arranged in line, and the width of the periodontal membrane is normal. Periodontal ligament fibroblasts set close, also neatly arranged and uniformly distributed between the main fibers. The direction is consistent with the long axis of the main fiber. The surface of alveolar bone is smooth, and there are no obvious signs of osteogenesis and bone absorption.

**1 d group**: On the pressure side: Periodontal membrane became narrow, collagen fibers arranged closely, the surface of alveolar bone was rough and there were 1-2 osteoclast in the resorption lacunae on the bone surface.

On the tension side: Periodontal membrane became broad, the collagen fibers were stretched and arranged loosely, with dilated blood vessels.

**3 d group**: On the pressure side: Periodontal membranes continue to become narrow, hyalinization began to appear. There were resorption lacunae on the bone surface, within the cellular structure is not clear, karyopyknosis are observed, some disappeared, while bone absorption is obvious, many osteoblasts were observed in lacunae.

On the tension side: Periodontal membranes continue to widen, and the periodontal membrane fibers are arranged disorderly, with more dilated and congested blood vessels.

**7 d group**: On the pessure side: Bone resorption lacunae increased, within a large number of multinucleated osteoclasts. Hyaline variable region became narrow and alveolar bone absorption was more obvious.

On the tension side: periodontal membrane fibers were disarranged and thickened. More blood vessels dilated and congested. Cuboidal osteoblasts appear on the alveolar surface of tension zone, the thickness of new bone deposition increases.

**2 w group**: On the pressure side: We observed an increase in the number of fibroblasts, and osteoclasts were still visible on the alveolar bone surface, extensive bone resorption was found. At the same time, more bone resorption lacunae appeared on the dental cervix and root furcation.

On the tension side: periodontal membrane fibers were arranged regularly and the thickness of new bone deposition is markedly thickened. We can see new bone line and osteoblasts which arranged neatly along the surface of the newly formed bone.

**4 w group**: On the pressure side: the number of osteoclasts decreased, periodontal tissue are being repaired, new bone deposition was visible in the bone resorption lacunae.

On the tension side: rows of osteoblasts and new bone or osteoid formation could be observed.

Figures:


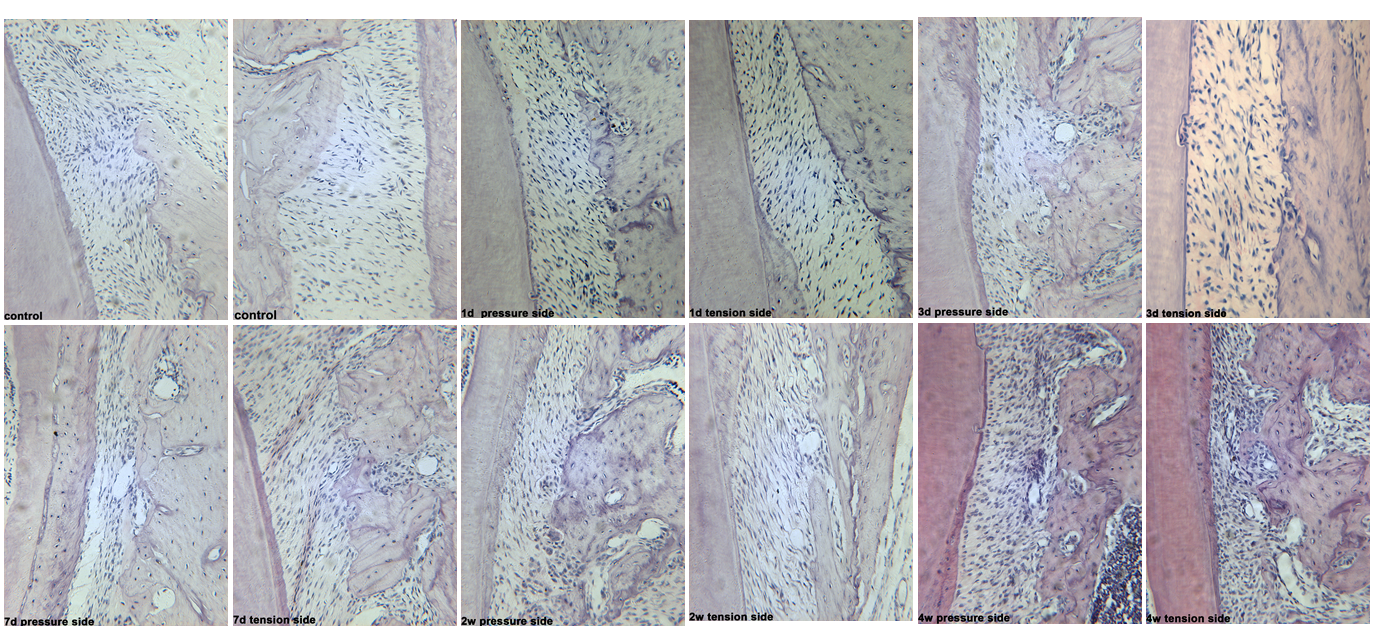


Histology of PL in control group and experimental group (HE, 200x)
